# Cryptic Diversity of *Isaria*-like Species in Guizhou, China

**DOI:** 10.3390/life11101093

**Published:** 2021-10-15

**Authors:** Wanhao Chen, Jiandong Liang, Xiuxiu Ren, Jiehong Zhao, Yanfeng Han, Zongqi Liang

**Affiliations:** 1 Center for Mycomedicine Research, Basic Medical School, Guizhou University of Traditional Chinese Medicine, Guiyang 550025, China; chenwanhao052@gzy.edu.cn (W.C.); jdliang317@gzy.edu.cn (J.L.); renxiuxiu207@gzy.edu.cn (X.R.); zhaojiehong020@gzy.edu.cn (J.Z.); 2 College of Ecological Engineering, Guizhou University of Engineering Science, Bijie 551700, China; 3 Institute of Fungus Resources, Department of Ecology, College of Life Sciences, Guizhou University, Guiyang 550025, China; zqliang472@126.com

**Keywords:** cryptic diversity, intraspecific, *Isaria*-like, multi-gene analysis

## Abstract

Many *Isaria*-like species have recently been moved into more appropriate genera. However, more robust molecular phylogenetic analyses are still required for *Isaria*-like fungi to ensure accurate taxonomic identification. We analyzed these *Isaria*-like strains using multi-gene phylogenetics. Cryptic diversity was discovered in several *Isaria farinosa* strains, and two new species, *Samsoniella pseudogunnii* and *S. pupicola*, are proposed. Our results reveal that more attention needs to be paid to cryptic intraspecific diversity across different isolates and genotypes of the *Isaria*-like species, some of which will need to be transferred to *Samsoniella*. Interestingly, *S. hepiali*, with a very broad host distribution, has been widely used as a medicinal and edible cordycipitoid fungus.

## 1. Introduction

The genus *Isaria* was originally establish based on the species *Isaria terrestris* Fr. [1]. Brown and Smith [2] transferred some species described in *Isaria* Pers. and *Spicaria* Harting into *Paecilomyces*, which possess a conidiogenous structure similar to that of *Paecilomyces variotii* Bainier. de Hoog [3] redescribed the genus *Isaria* and chose *Isaria felina* (DC.) Fr. as the lectotype. Typical characteristics include denticulate conidiogenous cells without elongation that arise in clusters from subtending cells or are solitarily from undifferentiated hyphae; mostly present synnemata; and globose, ellipsoidal, or subcylindrical conidia, mostly with a rounded base [3]. Samson [4] divided the genus *Paecilomyces* into two sections and all entomogenous species were placed in the section *Isarioidea*. Hodge et al. [5] reintroduced the genus *Isaria* with the type species *Isaria farinosa* (Holmsk.) Fr. and most entomopathogenic mesophilic *Paecilomyces* species were transferred to *Isaria* (Hypocreales, Clavicipitaceae) [6,7,8].

Kepler et al. [9] proposed the rejection of *Isaria* in favor of *Cordyceps* and transferred *Isaria* species into *Cordyceps*. Mongkolsamrit et al. [10] introduced some *Isaria*-like species and the new genus *Samsoniella* Mongkols., Noisrip., Thanakitp., Spatafora, and Luangsa-ard. Chen et al. [11,12] reported four *Isaria*-like species: *Akanthomyces araneogenus* Z.Q. Liang, W.H. Chen, and Y.F. Han; *Samsoniella coleopterorum* W.H. Chen, Y.F. Han, and Z.Q. Liang; *Samsoniella hymenopterorum* W.H. Chen, Y.F. Han, and Z.Q. Liang; and *Samsoniella lepidopterorum* W.H. Chen, Y.F. Han, and Z.Q. Liang. Currently, many species previously placed in the genus *Isaria* have been transferred to more appropriate genera. However, robust molecular phylogenetic analyses are still needed for *Isaria*-like fungi to ensure accurate taxonomic identification with comparable results across different isolates and genotypes [10].

We previously collected many *Isaria*-like morphs of invertebrate-pathogenic fungi from Guizhou Province, China. Some demonstrated close phylogenetic relationships with *Isaria farinosa* (Holmsk.) Fr. based on the analysis of associated ITS sequences. In the present study, we applied multi-gene (ITS, LSU, RPB1, RPB2, TEF) phylogenetic analysis to reevaluate the taxonomic position of these strains, as well as the cryptic diversity among the different isolates of *I. farinosa*, and to describe new taxa to accommodate the cryptic diversity of *Isaria*-like fungi.

## 2. Materials and Methods

### 2.1. Fungal Materials and Identification

The strains used in this study were isolated from infected insect and spider specimens collected in different areas of Guizhou Province, China, including Dali Forest in Rongjiang County, Yaorenshan National Forest Park in Sandu County, Mount Fanjing in Yinjiang County, Tongmuling in Guiyang City, and Doupengshan in Duyun City. Isolation of strains was conducted as described by Chen et al. [13]. Fungal colonies emerging from specimens were isolated and cultured at 25 °C for 14 days under 12 h light/12 h dark conditions following protocols described by Zou et al. [14]. Accordingly, the living isolates were obtained. The specimens and the isolated strains were deposited in the Institute of Fungus Resources, Guizhou University (formally Herbarium of Guizhou Agricultural College; code, GZAC), Guiyang City, Guizhou, China.

Macroscopic and microscopic morphological characteristics of the fungal isolates were examined, especially for the arrangement, shape, and measurement of phialides and conidia, and also the growth rates of cultures incubated at 25 °C for 14 days were determined in Potato Dextrose Agar (PDA) (Potato powder 6%, Agar 20%, Glucose 20%, Beijing Solarbio Technology Co., Ltd., China). Hyphae and conidiogenous structures were mounted in lactophenol cotton blue or 20% lactate solution and observed with an optical microscope (OM, DM4 B, Leica, Germany).

### 2.2. DNA Extraction, Polymerase Chain Reaction Amplification and Nucleotide Sequencing

DNA extraction was carried out with a fungal genomic DNA extraction kit (DP2033, BioTeke Corporation) in accordance with Liang et al. [15]. The extracted DNA was stored at −20 °C. The amplification of the internal transcribed spacer (ITS) region, the large subunit ribosomal RNA (LSU) gene, the RNA polymerase II largest subunit 1 (RPB1), the RNA polymerase II largest subunit 2 (RPB2), and the translation elongation factor 1 alpha (TEF) by PCR was described by White et al. [16], Rakotonirainy et al. [17], Castlebury et al. [18], and van den Brink et al. [19], respectively. PCR reactions for five loci of all strains were performed in a total volume of 25 μL containing 12.5 μL 2× PowerTaq PCR Master Mix (Tiangen Biotech (Beijing) Co., LTD, China), 1 μL of each primer (10 μM), 1 μL of genomic DNA (20–100 ng), and 9.5 μL of sterile water. Primer sequence information is shown in Table 1. PCR products were purified and sequenced at Sangon Biotech (Shanghai) Co. The resulting sequences were submitted to GenBank (the accession number is shown in Table 2).

### 2.3. Sequence Alignment and Phylogenetic Analyses

Lasergene software (version 6.0, DNASTAR) was applied for the assembling and editing of DNA sequence in this study. The ITS, LSU, RPB1, RPB2, and TEF sequences were downloaded from GenBank, based on Kepler et al. [9], Mongkolsamrit et al. [10,19], Chen et al. [12], Wang et al. [20], and others selected on the basis of BLAST algorithm-based searches in GenBank (Table 2). A single gene data set was aligned and edited by MAFFT v7.037b [21] and MEGA6 [22]. Combined sequences of ITS, LSU, RPB1, RPB2, and TEF were performed by SequenceMatrix v.1.7.8 [23]. The combined datasets (ITS+LSU+RPB2+TEF) and (ITS+LSU+RPB1+RPB2+TEF) were used to determine the family placement of those strains in Hypocreales and the taxonomic position of strains and the cryptic diversity among the different isolates of *I. farinosa* in Cordycipitaceae

The combined genes were both analyzed using the Bayesian inference (BI) and maximum likelihood (ML) methods. For BI, the model was selected for Bayesian analysis by ModelFinder [24] in the software PhyloSuite [25]. A Markov Chain Monte Carlo (MCMC) algorithm was used to generate phylogenetic trees with Bayesian probabilities using MrBayes v.3.2 [26] for the combined sequence datasets. The Bayesian analysis resulted in 20,001 trees after 10,000,000 generations. The first 4000 trees, representing the burn-in phase of the analyses, were discarded, while the remaining 16,001 trees were used for calculating posterior probabilities in the majority rule consensus tree. After the analysis was finished, each run was examined using the program Tracer v1.5 [27] to determine burn-in, confirming that both runs had converged. ML analyses were constructed with RAxMLGUI [28]. The GTRGAMMA model was used for all partitions, in accordance with recommendations in the RAxML manual against the use of invariant sites.

## 3. Results

### 3.1. Phylogenetic Analyses

**Figure 1:**
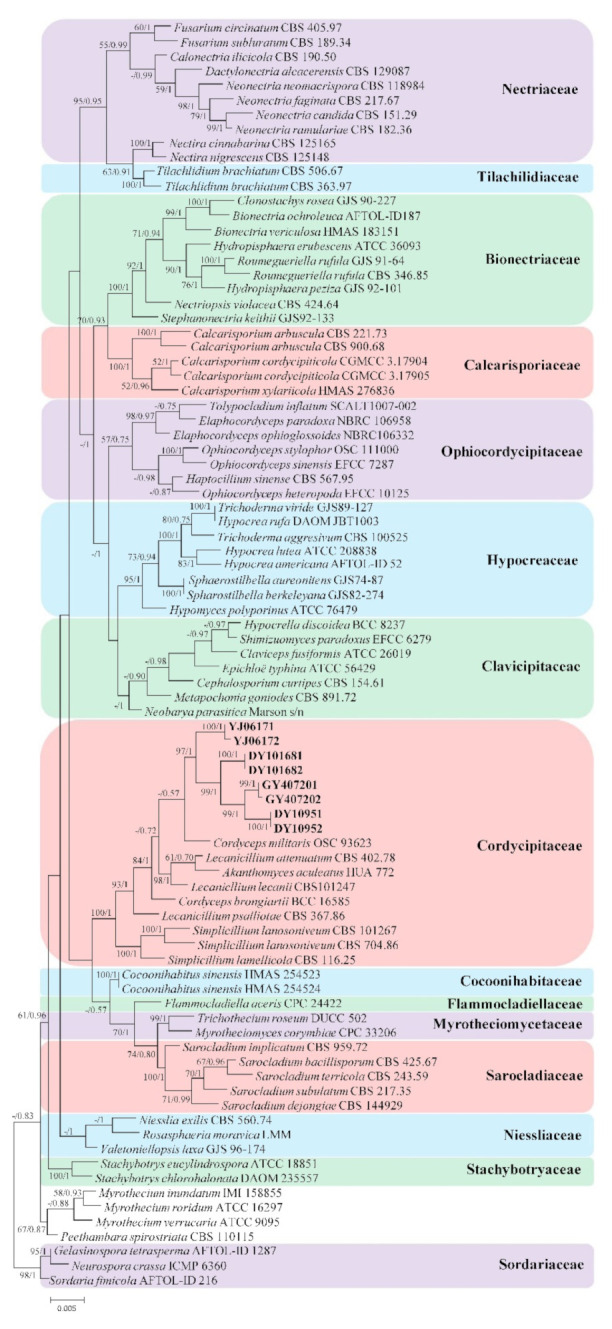
Phylogenetic placement of the new *Isaria*-like strains in the order of Hypocreales based on multigene dataset (ITS, LSU, RPB2m and TEF). Statistical support values (≥50%/0.5) are shown at the nodes for ML bootstrap support/BI posterior probabilities.

**Figure 2:**
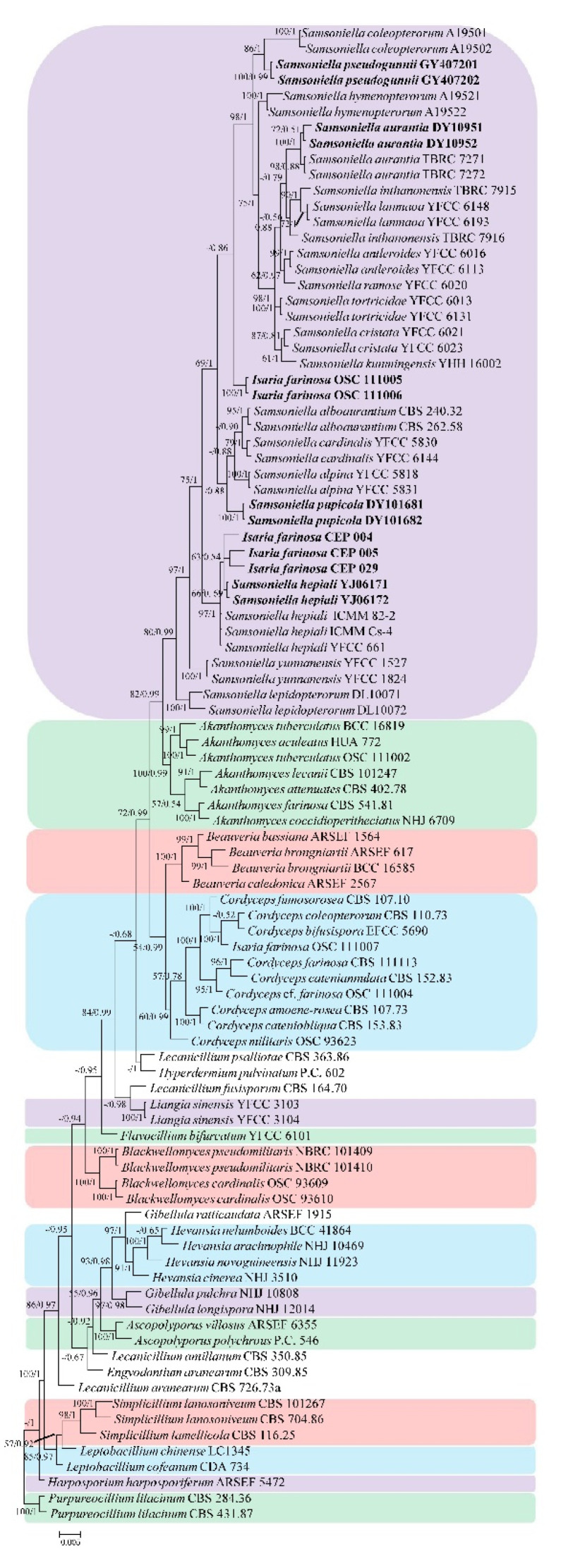
Phylogenetic placement of the new strains in Cordycipitaceae, based on multigene dataset (ITS, LSU, RPB1, RPB2, and TEF). Statistical support values (≥50%/0.5) are shown at the nodes for ML bootstrap support/BI posterior probabilities.

*Gelasinospora tetrasperma* Dowding, *Neurospora crassa* Shear and B.O. Dodge, and *Sordaria fimicola* (Roberge ex Desm.) Ces. and De Not. were used as the outgroup in analysis 1 (Figure 1) (to determine the family placement of those strains in Hypocreales). *Purpureocillium lilacinum* (Thom) Luangsa-ard, Houbraken, Hywel-Jones, and Samson was used as the outgroup in analysis 2 (Figure 2) (to determine the taxonomic position of strains and the cryptic diversity among the different isolates of *I. farinosa* in Cordycipitaceae). The concatenated sequences of analysis 1 and 2 included 77 and 62 taxa, respectively, and consisted of 2396 (ITS, 620; LSU, 712; RPB2, 510; and TEF, 554) and 3309 (ITS, 554; LSU, 677; RPB1, 533; RPB2, 671; and TEF, 874) characters with gaps, respectively.

Analysis 1: The selected models for BI analysis were GTR+F+I+G4 parameters for partition ITS and LSU+RPB2, and GTR+F+G4 parameters for partition TEF. The final value of the highest scoring tree was –37,321.078127, which was obtained from an ML analysis of the dataset (ITS+LSU+RPB2+TEF). The parameters of the general time reversible (GTR) model used to analyze the dataset were estimated using the following frequencies: A = 0.230263, C = 0.272892, G = 0.280445, and T = 0.216401; substitution rates AC = 1.451341, AG = 2.441940, AT = 1.532513, CG = 1.182477, CT = 5.701598, and GT = 1.000000; as well as the gamma distribution shape parameter α = 0.381402. In the phylogenetic tree (Figure 1), both analyses of ML and BI trees were largely congruent, and strongly supported in most branches. DY10951, DY10952, DY101681, DY101682, GY407201, GY407202, YJ06171, and YJ06172 strains had a close relationship with *Cordyceps* Fr., *Akanthomyces* Lebert, and *Simplicillium* W. Gams and Zare, and clustered into Cordycipitaceae.

Analysis 2: The selected models for BI analysis were GTR+F+I+G4 parameters for partition ITS+LSU+RPB2+TEF and GTR+F+G4 parameters for partition RPB1. The final value of the highest scoring tree was –31,206.916701, which was obtained from an ML analysis of the dataset (ITS+LSU+RPB1+RPB2+TEF). The parameters of the general time reversible (GTR) model used to analyze the dataset were estimated using the following frequencies: A = 0.238319, C = 0.279080, G = 0.271674, and T = 0.210926; substitution rates AC = 1.120096, AG = 2.745044, AT = 0.784066, CG = 0.934312, CT = 6.322628, and GT = 1.000000; as well as the gamma distribution shape parameter α = 0.308970. In the phylogenetic tree (Figure 2), both analyses of ML and BI trees were largely congruent, and strongly supported in most branches. The new strains were all clustered within the genus *Samsoniella*. GY407201 and GY407202 strains clustered with *Samsoniella coleopterorum* W.H. Chen, Y.F. Han, and Z.Q. Liang in a subclade. DY10951 and DY10952 strains clustered with *Samsoniella aurantia* Mongkols., Noisrip., Thanakitp., Spatafora, and Luangsa-ard in a subclade. DY101681 and DY101682 strains had a close relationship with *Samsoniella alboaurantia* (G. Sm.) Mongkols., Noisrip., Thanakitp., Spatafora, and Luangsa-ard; *Samsoniella alpina* H. Yu, Y.B. Wang, Y. Wang, and Zhu L. Yang; and *Samsoniella cardinalis* H. Yu, Y.B. Wang, Y. Wang, Q. Fan, and Zhu L. Yang. YJ06171 and YJ06172 strains clustered with *Isaria farinosa* (Holmsk.) Fr. in a subclade and had close relationship with *Samsoniella hepiali* (Q.T. Chen and R.Q. Dai ex R.Q. Dai, X.M. Li, A.J. Shao, Shu F. Lin, J.L. Lan, Wei H. Chen, and C.Y. Shen) H. Yu, R.Q. Dai, Y.B. Wang, Y. Wang, and Zhu L. Yang.

### 3.2. Taxonomy

#### 3.2.1. *Samsoniella pseudogunnii* W.H. Chen, Y.F. Han, J.D. Liang, and Z.Q. Liang, sp. nov.

MycoBank No.: MB840999

Etymology: referring to similar morphology with *Keithomyces neogunnii*.

Holotype: CHINA, Guizhou, Guiyang, Tongmuling (N26°23’, E106°40’). On a larva (Lepidoptera), 1 April 2019, Wanhao Chen, GZAC GY40720 (holotype), ex-type living cultures, GY407201, GY407202.

Description: Colonies on PDA, 4.1–4.3 cm diam. after 14 d at 25°C, white, consisting of a basal felt and cottony, floccose hyphal overgrowth, reverse yellowish. Prostrate hyphae smooth, septate, hyaline, 1.0–1.3 μm diam. Erect conidiophores usually arises from aerial hyphae. Phialides are solitary or in whorls of two to nine. Phialides 6.8–11.0 × 2.2–2.4 μm, with a cylindrical basal portion, tapering into a short distinct neck. Conidia in chains, hyaline, fusiform, one-celled, 2.8–3.2 × 1.7–2.1 μm. Chlamydospores and sexual state were not observed. Sizes and shapes of phialides and conidia are similar in culture and on natural substratum.

Known distribution: Tongmuling, Guiyang, Guizhou Province, China.

**Figure 3:**
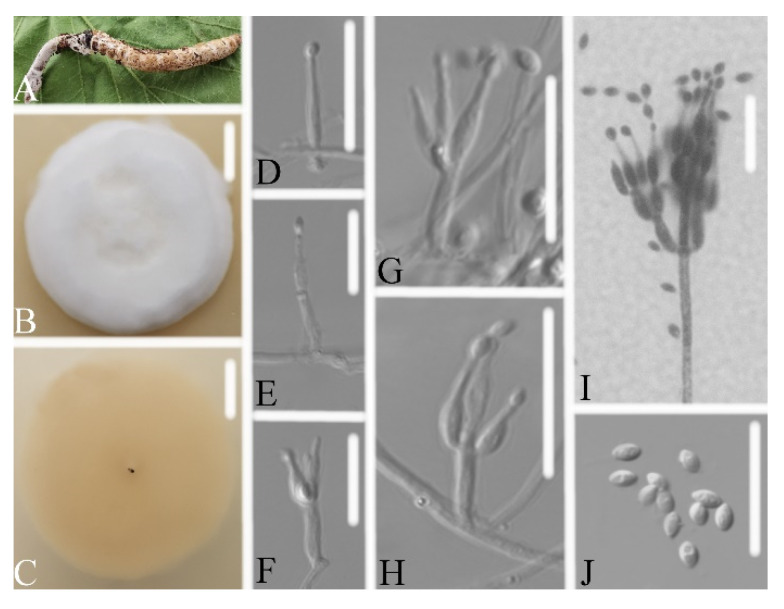
*Samsoniella pseudogunnii* (**A**) infected larva (Lepidoptera) (**B**,**C**) culture plate, showing the front (**B**) and the reverse (**C**) of the colony, cultured on PDA medium (**D**–**I**) phialides solitary, conidia adhering ellipsoidal slimy head and conidia (**J**) conidia. Scale bars: 10 mm (**B**,**C**), 10 μm (**D**–**J**).

Notes: *Samsoniella pseudogunnii* was identified as belonging to *Samsoniella* based on the phylogenetic analyses (Figure 2) and has a close relationship with *S. coleopterorum*. However, *Samsoniella pseudogunnii* (Figure 3) has longer phialide, larger conidia, and its larva host belongs to the order Lepidoptera.

#### 3.2.2. Samsoniella Pupicola W.H. Chen, Y.F. Han, J.D. Liang, and Z.Q. Liang, sp. nov.

MycoBank No.: MB841003

Etymology: referring to its pupa-inhabitor.

Holotype: CHINA, Guizhou, Qiannan Buyi and Miao Autonomous Prefecture, Duyun City (26°21′24.71″ N, 107°22′48.22″ E). On a pupa (Lepidoptera), 1 October 2019, Wanhao Chen, GZAC DY10168 (holotype), ex-type living cultures, DY101681, DY101682.

Description: Colonies on PDA, 2.3–2.4 cm diam. after 14 d at 25°C, white, consisting of a basal felt and cottony, floccose hyphal overgrowth, reverse yellowish. Prostrate hyphae smooth, septate, hyaline, 1.2–2.2 μm diam. Erect conidiophores usually arise from aerial hyphae. Phialides are solitary or in whorls of two to nine. Phialides 7.0–9.2 × 2.5–3.3 μm, with a cylindrical basal portion, tapering into a short distinct neck. Conidia in chains, hyaline, fusiform, one-celled, 2.5–3.3 × 2.2–2.6 μm. Chlamydospores and sexual state were not observed. Sizes and shapes of phialides and conidia are similar in culture and on natural substratum.

Known distribution: Duyun City, Qiannan Buyi and Miao Autonomous Prefecture, Guizhou Province, China.

Additional specimens examined: CHINA, Guizhou, Qiandongnan Miao and Dong Autonomous Prefecture, Rongjiang County (26°01′58.70″ N, 108°24′48.06″ E), on a lepidopteran pupa, 1 October 2018, W.H. Chen, GZAC DL1014.

**Figure 4:**
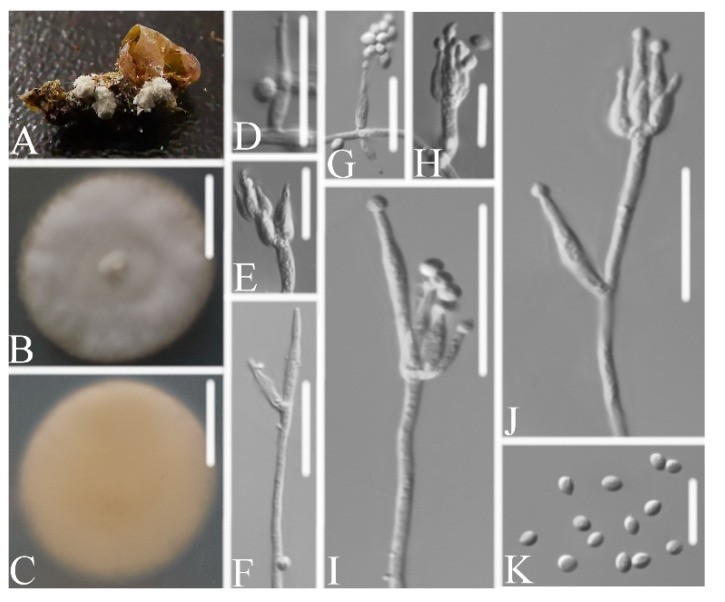
*Samsoniella pupicola* (**A**) infected pupa (Lepidoptera) (**B**,**C**) culture plate, showing the front (**B**) and the reverse (**C**) of the colony, cultured on PDA medium (**D**–**J**) phialides solitary, conidia adhering ellipsoidal slimy head and conidia (**K**) conidia. Scale bars: 10 mm (**B**,**C**), 10 μm (**D**–**K**).

Notes: *Samsoniella pupicola* was identified as belonging to *Samsoniella*, based on the phylogenetic analyses (Figure 2) and has a close relationship with *S. alboaurantium*, *S. alpina*, and *S. cardinalis*. However, *S. pupicola* (Figure 4) is distinguished from *S. alboaurantium* by having larger fusiform conidia, distinguished from *S. alpina* by having white colony and fusiform conidia, and distinguished from *S. cardinalis* by having shorter phialides.

#### 3.2.3. *Samsoniella aurantia* Mongkols., Noisrip., Thanakitp., Spatafora, and Luangsa-ard, Mycologia 110(1): 249 

Description: Colonies on PDA, 3.7–4.2 cm diam. after 14 d at 25°C, white, consisting of a basal felt and cottony, floccose hyphal overgrowth, pale green and pale pink in the middle of the colony, reverse yellowish and pale brown in the middle. Prostrate hyphae smooth, septate, hyaline, 1.3–2.6 μm diam. Erect conidiophores usually arise from aerial hyphae. Phialides are solitary or in whorls of two to ten. Phialides 3.6–7.7 × 1.3–1.6 μm, with a cylindrical basal portion, tapering into a short distinct neck. Conidia in chains, hyaline, fusiform, occasionally cylindrical, one-celled, 2.6–3.9 × 1.7–2.2 μm. Chlamydospores and sexual state were not observed. Sizes and shapes of phialides and conidia are similar in culture and on natural substratum.

Specimens examined: CHINA, Guizhou, Qiannan Buyi and Miao Autonomous Prefecture, Duyun City (26°21′24.71″ N, 107°22′48.22″ E). On a pupa (Lepidoptera), 1 October 2019, Wanhao Chen, GZAC DY1095, living cultures, DY10951, DY10952.

**Figure 5:**
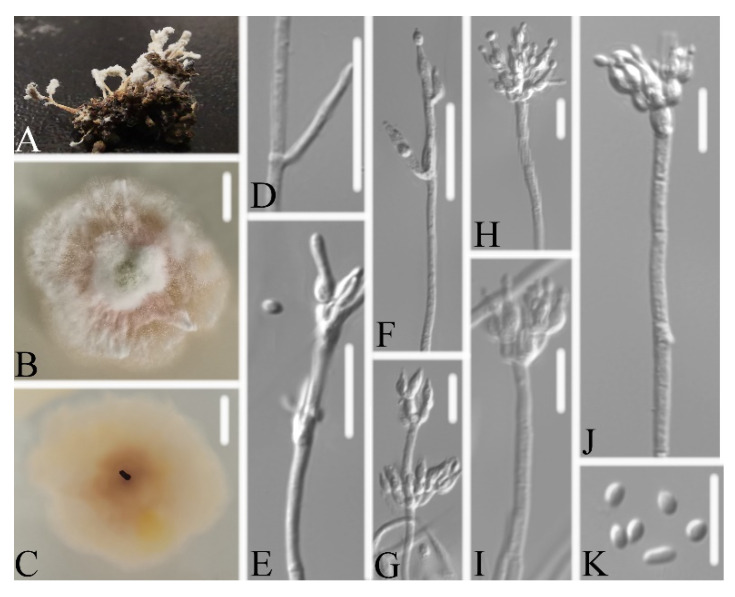
*Samsoniella aurantia* (**A**) infected pupa (Lepidoptera) (**B**,**C**) culture plate, showing the front (**B**) and the reverse (**C**) of the colony, cultured on PDA medium (**D**–**J**) phialides solitary, conidia adhering ellipsoidal slimy head and conidia (**K**) conidia. Scale bars: 10 mm (**B**,**C**), 10 μm (**D**–**K**).

Note: DY10951 and DY10952 strains were identified as belonging to *Samsoniella*, based on the phylogenetic analyses (Figure 2), and clustered with *Samsoniella aurantia* in a clade. The characteristics of DY10951 and DY10952 (Figure 5) strains are similar to that of *S. aurantia*, which had fusiform conidia (2–4 × 1–2 μm) and larger phialide (5–13 × 2–3 μm). Besides, the pairwise dissimilarities of ITS sequences show no difference within 554 bp between DY10951 and *S. aurantia*. Thus, molecular phylogenetic results and morphologically based conclusions support the idea that DY10951 and DY10952 strains were *S. aurantia*.

#### 3.2.4. *Samsoniella hepiali* (Q.T. Chen, and R.Q. Dai ex R.Q. Dai, X.M. Li, A.J. Shao, Shu F. Lin, J.L. Lan, Wei H. Chen, and C.Y. Shen) H. Yu, R.Q. Dai, Y.B. Wang, Y. Wang, and Zhu L. Yang, Fungal Diversity 103: 31

Description: Colonies on PDA, 5.8–5.9 cm diam. after 14 d at 25°C, white, consisting of a basal felt and cottony, floccose hyphal overgrowth, reverse yellowish. Prostrate hyphae smooth, septate, hyaline, 1.1–1.8 μm diam. Erect conidiophores usually arise from aerial hyphae. Phialides are solitary or in whorls of two to eight. Phialides 6.0–7.8 × 1.5–1.8 μm, with a cylindrical basal portion, tapering into a short distinct neck. Conidia in chains, hyaline, fusiform, one-celled, 2.1–2.5 × 0.9–1.6 μm. Chlamydospores and sexual state were not observed. Sizes and shapes of phialides and conidia are similar in culture and on natural substratum.

Specimens examined: CHINA, Guizhou, Tongren City, Yinjiang (N 27°55′17.1″, E 108°41′25.2″), on an ant, 1 October 2019, Wanhao Chen, GZAC YJ0617, DY1044, living cultures, YJ06171, YJ06172.

**Figure 6:**
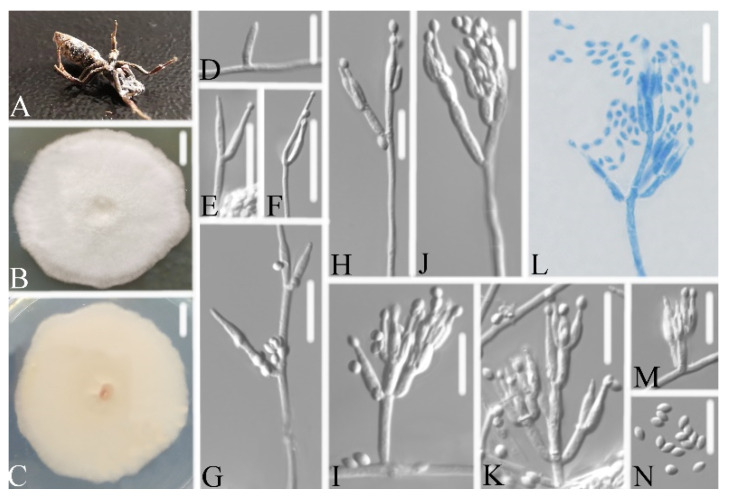
*Samsoniella hepiali* (**A**) infected ant (Formicidae) (**B**,**C**) culture plate, showing the front (**B**) and the reverse (**C**) of the colony, cultured on PDA medium (**D**–**J**) phialides solitary, conidia adhering ellipsoidal slimy head and conidia (**N**) conidia. Scale bars: 10 mm (**B**,**C**), 10 μm (**D**–**N**).

Note: YJ06171 and YJ06172 strains were identified as belonging to *Samsoniella*, based on the phylogenetic analyses (Figure 2), and clustered with *Samsoniella hepiali* in a clade. The characteristics of YJ06171 and YJ06172 (Figure 6) strains were very closely linked with *S. hepiali*, which had fusiform or oval conidia (1.8–3.3 × 1.4–2.2 μm) and larger phialide (3.5–13.6 × 1.3–2.1 μm). Besides, the pairwise dissimilarities of LSU sequences show no difference within 677 bp between YJ06171 and *S. hepiali*. Thus, molecular phylogenetic results and morphologically based conclusions supported the idea that YJ06171 and YJ06172 strains were *S. hepiali*.

## 4. Discussion

The taxonomic delimitation of *Isaria* was originally based on morphological characteristics. However, *Isaria* shares many morphological characters with other genera in Hypocreales, which has resulted in a turbulent taxonomic history [10,29]. D’Alessandro et al. [30] noted that the morphological characteristics used to classify the genus *Isaria* frequently do not resolve new isolates into clearly defined species and need additional molecular markers in phylogenetic analyses. In the present study, *Isaria*-like strains collected from Guizhou Province, China, and previously identified by morphological characteristics, were reanalyzed using multi-gene (ITS, LSU, RPB1, RPB2, TEF) phylogenetic methodology. We proposed two new species of *Samsoniella* in this study.

The species *Isaria farinosa* is a well-known entomopathogenic fungi with worldwide distribution and a wide host range [31]. Kepler et al. [9] transferred *Isaria farinosa* to the genus *Cordyceps* as *C. farinosa* (Holmsk.) Kepler, B. Shrestha, and Spatafora based on a phylogenetic analysis of the CBS 111113 strain. We analyzed several strains of *Isaria farinosa* in the present study. Some properly belonged in the genus *Samsoniella*. CEP 004, CEP 005, CEP 029, YJ06171, and YJ06172 strains were identified as *S. hepiali*. Strains DY10951 and DY10952 were identified as *S. aurantia*. OSC 111005 and OSC 111006 strains were identified as new species but are absent in delineating morphological characteristics. Our results reveal cryptic diversity present in *Isaria farinosa* (now treated as *Cordyceps farinosa*) and illustrated that more attention should be paid on cryptic intraspecific diversity across different fungi isolates and genotypes.

The genus *Samsoniella* was established for the typical species *S. inthanonensis* Mongkolsamrit, Noisripoom, Thanakitpipattana, Spatafora, and Luangsa-ard, and two other species (*S. alboaurantia* (G. Sm.) Mongkols., Noisrip., Thanakitp., Spatafora, and Luangsa-ard and *S. aurantia* Mongkols., Noisrip., Thanakitp., Spatafora, and Luangsa-ard) [10]. *Samsoniella* species all have *Isaria*-like morphological characteristics, and cluster in an independent clade with close relationship to the genus *Akanthomyces*. The species *S. alboaurantia* was established based on two strains, CBS 240.32 and CBS 262.58, which previously belonged to *Isaria farinosa* (originally designated *Paecilomyces farinosus* (Holmsk.) A.H.S. Br. and G. Sm.) [10]. Lin et al. [32] revised the taxonomy of some *Isaria*-like strains originally identified as *Isaria farinosa* by morphological characteristics using multi-gene phylogenetic analysis. All the strains were identified as *Samsoniella hepiali* (Q.T. Chen and R.Q. Dai ex R.Q. Dai, X.M. Li, A.J. Shao, Shu F. Lin, J.L. Lan, Wei H. Chen, and C.Y. Shen) H. Yu, R.Q. Dai, Y.B. Wang, Y. Wang, and Zhu L. Yang. In the present study, YJ06171 and YJ06172 strains were also identified as *Samsoniella hepiali*. Our results revealed that more isolates and genotypes, originally designated as *Isaria*, will need to be transferred to *Samsoniella*.

*Samsoniella hepiali* (otherwise known as *Paecilomyces hepiali*) is isolated from a field collection of natural *Ophiocordyceps sinensis* insect–fungi complex [33], and is widely used as a medicinal and edible cordycipitoid fungus, creating a great economic value [20]. Lin et al. [32] reported six isolates of *Samsoniella hepiali* from Anhui Province, China, which were isolated from leafhopper, larva, and cicada. CEP 004, CEP 005, CEP 029 strains from Buenos Aires, Argentina, were isolated from whitefly and soil [30]. YJ06171 and YJ06172 strains from Guizhou Province, China were isolated from ant. It is interesting that *Samsoniella hepiali* and its hosts are widely distributed in China and Argentina. This result will help us to assess the extent and distribution of genetic diversity of *Samsoniella hepiali* on a large scale, understand its biology and demographic history, and guide biodiversity conservation programs.

## Figures and Tables

**Table 1:** Primers information for 5-locus DNA sequences.

Name		Length	Direction	Sequence 5′-3′	Optimised PCR Protocols	References
ITS	ITS5	22	forward	GGAAGTAAAAGTCGTAACAAGG	(95 °C: 30 s, 51 °C: 50 s, 72 °C: 45 s) × 33 cycles	[16]
	ITS4	20	reverse	TCCTCCGCTTATTGATATGC		
LSU	LROR	17	forward	ACCCGCTGAACTTAAGC	(94 °C: 30 s, 51 °C: 1 min, 72 °C: 2 min) × 33 cycles	[17]
	LR5	17	reverse	TCCTGAGGGAAACTTCG		
RPB1	CRPB1	20	forward	CAYCCWGGYTTYATCAAGAA	(94 °C: 30 s, 55 °C: 30 s, 72 °C: 1 min) × 33 cycles	[18]
	RPB1Cr	23	reverse	CCNGCDATNTCRTTRTCCATRTA		
RPB2	RPB2-5F3	20	forward	GACGACCGTGATCACTTTGG	(94 °C: 30 s, 54 °C: 40 s, 72 °C: 1 min 20 s) × 33 cycles	[19]
	RPB2-7Cr2	20	reverse	CCCATGGCCTGTTTGCCCAT		
TEF	983F	23	forward	GCYCCYGGHCAYCGTGAYTTYAT	(94 °C: 30 s, 58 °C: 1 min 20 s, 72 °C: 1 min) × 33 cycles	[19]
	2218R	23	reverse	ATGACACCRACRGCRACRGTYTG		

**Table 2:** List of strains and GenBank accession numbers of sequences used in this study.

Species	Strain No.	Host/ Substratum	GenBank Accession No.				
			ITS	LSU	RPB1	RPB2	TEF
*Akanthomyces aculeatus*	HUA 772	Lepidoptera; Sphingidae	-	KC519370	-	-	KC519366
*A. attenuates*	CBS 402.78	Leaf litter (*Acer saccharum*)	-	AF339565	EF468888	EF468935	EF468782
*A. coccidioperitheciatus*	NHJ 6709	Araneae (Spider)	-	EU369042	EU369067	-	EU369025
*A. farinosa*	CBS 541.81	-	AY624180	MF416553	MF416655	MF416449	JQ425686
*A. tuberculatus*	BCC 16819	Lepidoptera (Adult moth)	-	GQ249987	-	-	GQ250037
*A. tuberculatus*	OSC 111002	Lepidoptera	-	DQ518767	DQ522384	-	DQ522338
*Ascopolyporus polychrous*	P.C. 546	Plant	-	DQ118737	DQ127236	-	DQ118745
*A. villosus*	ARSEF 6355	Plant	-	AY886544	DQ127241	-	DQ118750
*Beauveria bassiana*	ARSEF 1564	Lepidoptera; Arctiidae	-	-	HQ880833	HQ880905	HQ880974
*B. brongniartii*	ARSEF 617	Coleoptera; Scarabaeidae	-	AB027381	HQ880854	HQ880926	HQ880991
*B. brongniartii*	BCC 16585	Coleoptera (*Anomala cuprea*)	JN049867	JF415967	JN049885	JF415991	JF416009
*B. caledonica*	ARSEF 2567	Soil	-	AF339520	HQ880889	HQ880961	EF469057
*Bionectria ochroleuca*	AFTOL-ID187	-	-	DQ862027	-	DQ862013	DQ862029
*B. vericulosa*	HMAS 183151	Plant	HM050304	HM050302	-	-	-
*Blackwellomyces cardinalis*	OSC 93609	Lepidoptera; Tineidae (Larva)	-	AY184962	DQ522370	-	DQ522325
*B. cardinalis*	OSC 93610	Lepidoptera; Tineidae (Larva)	-	AY184963	EF469088	-	EF469059
*B. pseudomilitaris*	NBRC 101409	Lepidoptera (Larva)	-	JN941393	JN992482	-	-
*B. pseudomilitaris*	NBRC 101410	Lepidoptera (Larva)	-	JN941394	JN992481	-	-
*Calcarisporium arbuscula*	CBS 221.73	-	AY271809	-	-	-	-
*C. arbuscula*	CBS 900.68	Hymenomycetes (*Agarics* sp.)	KT945003	KX442598	-	KX442597	KX442596
*C. cordycipiticola*	CGMCC 3.17904	Cordycipitaceae (*Cordyceps militaris*)	KT945001	KX442604	-	KX442607	KX442605
*C. cordycipiticola*	CGMCC 3.17905	Cordycipitaceae (*Cordyceps militaris*)	KT944999	KX442599	-	KX442594	KX442593
*C. xylariicola*	HMAS 276836	Xylariaceae (*Xylaria* sp.)	KX442603	KX442601	-	KX442606	KX442595
*Calonectria ilicicola*	CBS 190.50	Plant	GQ280605	GQ280727	-	KM232307	AY725726
*Cephalosporium curtipes*	CBS 154.61	Uredinales (*Hemileia vastatrix*)	AJ292404	AF339548	-	EF468947	EF468802
*Claviceps fusiformis*	ATCC 26019	Poaceae	JN049817	-	-	-	DQ522320
*Clonostachys rosea*	GJS 90-227	Plant	-	AY489716	-	-	AY489611
*Cocoonihabitus sinensis*	HMAS 254523	Saturniidae (Cocoon)	KY924870	KY924869	-	-	-
*C. sinensis*	HMAS 254524	Saturniidae (Cocoon)	MF687395	MF687396	-	-	-
*Cordyceps amoene-rosea*	CBS 107.73	Coleoptera (Pupa)	MH860646	MH872342	MF416651	-	-
*C. bifusispora*	EFCC 5690	Lepidoptera (Pupa)	-	EF468806	EF468854	EF468909	EF468746
*C. cateniannulata*	CBS 152.83	Coleoptera (Adult)	NR_111169	NG_067333	-	-	-
*C. cateniobliqua*	CBS 153.83	Lepidoptera (*Adoxophyesprivatana*)	NR_111170	-	-	-	JQ425688
*C.* cf. *farinosa*	OSC 111004	Lepidoptera (Pupa)	-	EF468840	EF468886	-	EF468780
*C. coleopterorum*	CBS 110.73	Coleoptera (Larva)	AY624177	JF415988	JN049903	JF416006	JF416028
*C. farinosa*	CBS 111113	-	-	MF416554	MF416656	MF416450	MF416499
*C. fumosorosea*	CBS 107.10	-	-	MF416556	MF416659	MF416453	MF416502
*C. militaris*	OSC 93623	Lepidoptera (Pupa)	-	AY184966	DQ522377	AY545732	DQ522332
*Dactylonectria alcacerensis*	CBS 129087	Plant (*Vitis vinifera*)	JF735333	KM231629	-	-	JF735819
*Elaphocordyceps ophioglossoides*	NBRC106332	-	JN943322	JN941409	-	-	-
*E. paradoxa*	NBRC 106958	-	JN943324	JN941411	-	-	-
*Engyodontium aranearum*	CBS 309.85	Araneae (Spider)	-	AF339526	DQ522387	DQ522439	DQ522341
*Epichloë typhina*	ATCC 56429	Poaceae (*Festuca rubra*)	JN049832	U17396	-	DQ522440	AF543777
*Flammocladiella aceris*	CPC 24422	Plant (*Acer platanoides*)	KR611883	KR611901	-	-	-
*Flavocillium bifurcatum*	YFCC 6101	Noctuidae (Larva)	-	MN576781	MN576841	MN576897	MN576951
*Fusarium circinatum*	CBS 405.97	-	U61677	-	-	JX171623	KM231943
*F. subluratum*	CBS 189.34	Soil	HQ897830	KM231680	-	-	-
*Gelasinospora tetrasperma*	AFTOL-ID 1287	-	-	DQ470980	-	DQ470932	DQ471103
*Gibellula longispora*	NHJ 12014	Araneae (Spider)	-	-	EU369055	-	EU369017
*G. pulchra*	NHJ 10808	Araneae (Spider)	-	EU369035	EU369056	-	EU369018
*G. ratticaudata*	ARSEF 1915	Araneae (Spider)	-	DQ518777	DQ522408	-	DQ522360
*Haptocillium sinense*	CBS 567.95	Nematode	AJ292417	AF339545	-	-	-
*Harposporium harposporiferum*	ARSEF 5472	-	-	NG_060621	-	-	-
*Hevansia arachnophile*	NHJ 10469	Araneae (Spider)	-	EU369031	EU369047	-	EU369008
*H. cinerea*	NHJ 3510	Araneae (Spider)	-	-	EU369048	-	EU369009
*H. nelumboides*	BCC 41864	Araneae (Spider)	JN201871	JN201873	-	-	JN201867
*H. novoguineensis*	NHJ 11923	Araneae (Spider)	-	EU369032	EU369052	-	EU369013
*Hyperdermium pulvinatum*	P.C. 602	Hemiptera (Scale insect)	-	DQ118738	DQ127237	-	DQ118746
*Hydropisphaera erubescens*	ATCC 36093	-	-	AF193230	-	AY545731	DQ518174
*H. peziza*	GJS 92-101	Plant (Bark)	-	AY489730	-	-	AY489625
*Hypocrea americana*	AFTOL-ID 52	-	DQ491488	AY544649	-	-	DQ471043
*H. lutea*	ATCC 208838	On decorticated conifer wood	-	AF543791	-	DQ522446	AF543781
*H. rufa*	DAOM JBT1003	-	JN942883	JN938865			
*H. discoidea*	BCC 8237	-	JN049840	DQ384937	-	DQ452461	DQ384977
*Hypomyces polyporinus*	ATCC 76479	-	-	AF543793	-	-	AF543784
*Isaria farinosa*	CEP 004	Soil	JN998783	-	-	-	JN998763
*I. farinosa*	CEP 005	Soil	JN998784	-	-	-	JN998764
*I. farinosa*	CEP 029	*Trialeurodes vaporariorum*	JN998785	-	-	-	JN998765
*I. farinosa*	OSC 111005	Lepidoptera (Pupa)	-	DQ518772	DQ522394	-	DQ522348
*I. farinosa*	OSC 111006	Lepidoptera (Pupa)	-	EF469080	EF469094	-	EF469065
*I. farinosa*	OSC 111007	Lepidoptera (Pupa)	-	DQ518773	DQ522395	DQ522449	DQ522349
*Lecanicillium antillanum*	CBS 350.85	Hymenomycetes (*Agaric* sp.)	-	AF339536	DQ522396	DQ522450	DQ522350
*L. attenuatum*	CBS 402.78	Leaf litter of Acer saccharum	-	AF339565	EF468888	EF468935	EF468782
*L. aranearum*	CBS 726.73a	Arachnida (Spider)	-	AF339537	EF468887	EF468934	EF468781
*L. fusisporum*	CBS 164.70	Hymenomycetes (*Coltricia perennis*)	-	AF339549	EF468889	-	EF468783
*L. psalliotae*	CBS 367.86	*Puccinia graminis*	-	KM283800	-	-	KM283823
*L. lecanii*	CBS101247	Hemiptera (*Coccus viridis*)	JN049836	KM283794	-	KM283859	DQ522359
*Leptobacillium chinense*	LC 1345	submerged wood	-	JQ410322	-	-	-
*L. coffeanum*	CDA 734	Plant (*Coffea arabica*)	-	MF066032	-	-	-
*Liangia sinensis*	YFCC 3103	Fungi (*Beauveria yunnanensis*)	-	MN576782	MN576842	MN576898	MN576952
*L. sinensis*	YFCC 3104	Fungi (*Beauveria yunnanensis*)	-	MN576783	MN576843	MN576899	MN576953
*Metapochonia goniodes*	CBS 891.72	Fungi	AJ292409	AF339550	DQ522401	DQ522458	DQ522354
*Myrotheciomyces corymbiae*	CPC 33206	Plant (*Corymbia variegata*)	NR_160351	NG_064542	-	-	-
*Myrothecium inundatum*	IMI 158855	Hymenomycetes (*Russula nigricans*)	-	AY489731	-	-	AY489626
*M. roridum*	ATCC 16297	Soil	-	AY489708	-	-	AY489603
*M. verrucaria*	ATCC 9095	Plant (*Gossypium* sp.)	-	AY489713	-	-	AY489608
*Nectira cinnabarina*	CBS 125165	Plant (*Aesculus* sp.)	HM484548	HM484562	-	KM232402	HM484527
*N. nigrescens*	CBS 125148	Plant (Dicotyledonous tree)	HM484707	HM484720	-	KM232403	HM484672
*Nectriopsis violacea*	CBS 424.64	Fungi (*Fuligo* sp.)	-	AY489719	-	-	-
*Neobarya parasitica*	Marson s/n	Fungi (*Bertia moriformis*)	KP899626	KP899626	-	-	-
*Neonectria candida*	CBS 151.29	Plant (*Malus sylvestris*)	JF735313	AY677333	-	-	JF735791
*N. faginata*	CBS 217.67	-	HQ840385	HQ840382	-	DQ789797	JF268746
*N. neomacrispora*	CBS 118984	-	HQ840388	HQ840379	-	DQ789810	JF268754
*N. ramulariae*	CBS 182.36	-	HM054157	HM042435	-	DQ789793	HM054092
*Neurospora crassa*	ICMP 6360	-	AY681193	AY681158	-	-	-
*Niesslia exilis*	CBS 560.74	-	-	AY489720	-	-	AY489614
*Ophiocordyceps heteropoda*	EFCC 10125	Cicadidae (*Tibicen bihamatus*)	JN049852	EF468812	-	EF468914	EF468752
*O. sinensis*	EFCC 7287	Lepidoptera (Ghostmoth)	JN049854	EF468827	-	EF468924	EF468767
*O. stylophor*	OSC 111000	Insect (Larvae)	JN049828	DQ518766	-	DQ522433	DQ522337
*Peethambara spirostriata*	CBS 110115	Plant (*Theobroma cacao*)	-	AY489724	-	EF692516	AY489619
*Purpureocillium lilacinum*	CBS 284.36	Soil	-	AY624227	EF468898	EF468941	EF468792
*P. lilacinum*	CBS 431.87	Nematoda (*Meloidogyne* sp.)	HQ842812	EF468844	EF468897	EF468940	EF468791
*Rosasphaeria moravica*	LMM	-	JF440985	-	-	JF440986	JF440987
*Roumegueriella rufula*	GJS 91-64	-	-	EF469082	-	EF469116	EF469070
*R. rufula*	CBS 346.85	-	-	DQ518776	-	DQ522461	DQ522355
*Samsoniella alboaurantium*	CBS 240.32	Lepidoptera (Pupa)	-	JF415979	JN049895	JF415999	JF416019
*S. alboaurantium*	CBS 262.58	Soil	-	AB080087	MF416654	MF416448	MF416497
*S. alpina*	YFCC 5818	Hepialidae (*Hepialus baimaensis*)	-	MN576809	MN576869	MN576923	MN576979
*S. alpina*	YFCC 5831	Hepialidae (*Hepialus baimaensis*)	-	MN576810	MN576870	MN576924	MN576980
*S. antleroides*	YFCC 6016	Noctuidae (Larvae)	-	MN576803	MN576863	MN576917	MN576973
*S. antleroides*	YFCC 6113	Noctuidae (Larvae)	-	MN576804	MN576864	MN576918	MN576974
*S. aurantia*	TBRC 7271	Lepidoptera	-	MF140728	MF140791	MF140818	MF140846
*S. aurantia*	TBRC 7272	Lepidoptera	-	MF140727	-	MF140817	MF140845
*S. aurantia*	DY10951	Lepidoptera (Pupa)	MZ827667	MZ827827	-	-	MZ855229
*S. aurantia*	DY10952	Lepidoptera (Pupa)	MZ827666	MZ827084	-	-	MZ855230
*S. cardinalis*	YFCC 5830	Limacodidae (Pupa)	-	MN576788	MN576848	MN576902	MN576958
*S. cardinalis*	YFCC 6144	Limacodidae (Pupa)	-	MN576786	MN576846	MN576900	MN576956
*S. coleopterorum*	A19501	Curculionidae (Snout beetle)	MT626376	-	MT642600	MN101585	MN101586
*S. coleopterorum*	A19502	Curculionidae (Snout beetle)	MT626625	-	MT642603	MN101587	MT642602
*S. cristata*	YFCC 6021	Saturniidae (Pupa)	-	MN576791	MN576851	MN576905	MN576961
*S. cristata*	YFCC 6023	Saturniidae (Pupa)	-	MN576792	MN576852	MN576906	MN576962
*S. hepiali*	ICMM 82-2	Fungi (*Ophiocordyceps sinensis*)	-	MN576794	MN576854	MN576908	MN576964
*S. hepiali*	ICMM Cs-4	Fungi (*Ophiocordycepssinensis*)	-	MN576799	MN576859	MN576913	MN576969
*S. hepiali*	YFCC 661	Fungi (*Ophiocordycepssinensis*)	-	MN576795	MN576855	MN576909	MN576965
*S. hepiali*	YJ06171	Formicidae	MZ831866	MZ831868	MZ855241	-	MZ855235
*S. hepiali*	YJ06172	Formicidae	MZ831867	MZ831873	-	-	MZ855236
*S. hymenopterorum*	A19521	Vespidae (Bee)	MN128224	-	MT642601	MT642604	MN101588
*S. hymenopterorum*	A19522	Vespidae (Bee)	MN128081	-	MN101589	MN101590	MN101591
*S. inthanonensis*	TBRC 7915	Lepidoptera (Pupa)	MF140761	-	MF140790	MF140815	MF140849
*S. inthanonensis*	TBRC 7916	Lepidoptera (Pupa)	MF140760	-	MF140789	MF140814	MF140848
*S. kunmingensis*	YHH 16002	Lepidoptera (Pupa)	-	MN576802	MN576862	MN576916	MN576972
*S. lanmaoa*	YFCC 6148	Lepidoptera (Pupa)	-	MN576789	MN576849	MN576903	MN576959
*S. lanmaoa*	YFCC 6193	Lepidoptera (Pupa)	-	MN576790	MN576850	MN576904	MN576960
*S. lepidopterorum*	DL10071	Lepidoptera (Pupa)	MN128076	-	MN101592	MN101593	MN101594
*S. lepidopterorum*	DL10072	Lepidoptera (Pupa)	MN128084	-	-	MT642605	MT642606
*S. pseudogunii*	GY407201	Lepidoptera (Larvae)	MZ827470	MZ827010	-	MZ855239	MZ855233
*S. pseudogunii*	GY407202	Lepidoptera (Larvae)	MZ831863	MZ831865	-	MZ855240	MZ855234
*S. pupicola*	DY101681	Lepidoptera (Pupa)	MZ827085	MZ827009	-	MZ855237	MZ855231
*S. pupicola*	DY101682	Lepidoptera (Pupa)	MZ827008	MZ827635	-	MZ855238	MZ855232
*S. ramose*	YFCC 6020	Limacodidae (Pupa)	-	MN576805	MN576865	MN576919	MN576975
*S. tortricidae*	YFCC 6013	Limacodidae (Pupa)	-	MN576807	MN576867	MN576921	MN576977
*S. tortricidae*	YFCC 6131	Limacodidae (Pupa)	-	MN576806	MN576866	MN576920	MN576976
*S. yunnanensis*	YFCC 1527	Limacodidae (Pupa)	-	MN576812	MN576872	MN576926	MN576982
*S. yunnanensis*	YFCC 1824	Limacodidae (Pupa)	-	MN576813	MN576873	MN576927	MN576983
*Sarocladium bacillisporum*	CBS 425.67	Soil	NR_145039	MH870718	-	-	-
*S. dejongiae*	CBS 144929	Soil	NR_161153	NG_067854	-	-	-
*S. implicatum*	CBS 959.72	Soil	HG965023	MH878470	-	-	-
*S. subulatum*	CBS 217.35	Soil	MH855652	NG_070566	-	-	-
*S. terricola*	CBS 243.59	Soil	MH857853	MH869389	-	-	-
*Shimizuomyces paradoxus*	EFCC 6279	Smilacaceae (*Smilax sieboldii*)	JN049847	EF469084	-	EF469117	EF469071
*Simplicillium lamellicola*	CBS 116.25	Hymenomycetes (*Agaricus bisporus*)	AJ292393	MH866307	-	DQ522462	DQ522356
*S. lanosoniveum*	CBS 101267	Uredinales (*Hemileia vastatrix*)	-	AJ292395	-	DQ522463	DQ522357
*S. lanosoniveum*	CBS 704.86	Uredinales (*Hemileia vastatrix*)	AJ292396	AF339553	-	DQ522464	DQ522358
*Sordaria fimicola*	AFTOL-ID 216	-	DQ518178	-	-	-	DQ518175
*Sphaerostilbella aureonitens*	GJS74-87	-	FJ442633	HM466683	-	FJ442763	-
*S. berkeleyana*	GJS82-274	-	-	U00756	-	-	AF543783
*Stachybotrys chlorohalonata*	DAOM 235557	-	JN942888	JN938870	-	-	-
*S. eucylindrospora*	ATCC 18851	-	JN942887	JN938869	-	-	-
*Stephanonectria keithii*	GJS92-133	Plant (Bark)	-	AY489727	-	-	AY489622
*Tilachlidium brachiatum*	CBS 363.97	Hymenomycetes (*Agaricus* sp.)	KM231838	KM231719	-	KM232414	KM231975
*T. brachiatum*	CBS 506.67	Hymenomycetes (*Hypholoma fasciculare*)	KM231839	HQ232177	-	KM232415	KM231976
*Tolypocladium inflatum*	SCALT1007-002	Sclerotium	KC963032	-	-	-	-
*Trichoderma aggresivum*	CBS 100525	-	-	JN939837	-	JQ014130	-
*T. viride*	GJS89-127	Plant (Bark)	-	AY489726	-	-	AY489621
*Trichothecium roseum*	DUCC 502	Plant (*Solanum lycopersicum*)	JN937590	JX458860	-	-	-
*Valetoniellopsis laxa*	GJS 96-174	-	-	AY015635	-	AY015638	-

## Data Availability

Not applicable.
